# Population Differentiation and Species Formation in the Deep Sea: The Potential Role of Environmental Gradients and Depth

**DOI:** 10.1371/journal.pone.0077594

**Published:** 2013-10-01

**Authors:** Robert M. Jennings, Ron J. Etter, Lynn Ficarra

**Affiliations:** Biology Department, University of Massachusetts Boston, Boston, Massachusetts, United States of America; University of Massachusetts, United States of America

## Abstract

Ecological speciation probably plays a more prominent role in diversification than previously thought, particularly in marine ecosystems where dispersal potential is great and where few obvious barriers to gene flow exist. This may be especially true in the deep sea where allopatric speciation seems insufficient to account for the rich and largely endemic fauna. Ecologically driven population differentiation and speciation are likely to be most prevalent along environmental gradients, such as those attending changes in depth. We quantified patterns of genetic variation along a depth gradient (1600-3800m) in the western North Atlantic for a protobranch bivalve ( *Nuculaatacellana* ) to test for population divergence. Multilocus analyses indicated a sharp discontinuity across a narrow depth range, with extremely low gene flow inferred between shallow and deep populations for thousands of generations. Phylogeographical discordance occurred between nuclear and mitochondrial loci as might be expected during the early stages of species formation. Because the geographic distance between divergent populations is small and no obvious dispersal barriers exist in this region, we suggest the divergence might reflect ecologically driven selection mediated by environmental correlates of the depth gradient. As inferred for numerous shallow-water species, environmental gradients that parallel changes in depth may play a key role in the genesis and adaptive radiation of the deep-water fauna.

## Introduction

How species form is one of the most fundamental questions in evolutionary biology. Over the past two decades considerable progress has been made in identifying the scales, mechanisms, and driving forces of species formation in terrestrial and shallow-water ecosystems (e.g. [1-7]). However, little is known about these processes in the deep ocean, arguably the largest evolutionary realm on Earth with few obvious barriers to gene flow.

Geographic patterns of population genetic structure provide one of the primary lines of evidence for identifying the forces that might isolate gene pools. Marine organisms with pelagic dispersal were originally thought to disperse widely and show little population divergence [8], but recent empirical work has found that dispersal is much more constrained than typically inferred based on life histories (e.g. [2,9-11]). A number of mechanisms have been identified that might limit gene flow in marine ecosystems [3,12] including distance (isolation by distance – [13,14]), hydrographic features [15-17], nonrandom dispersal [18], gametic incompatibility systems [19-22], historical vicariance [23-27] and strong environmental gradients [28-33].

A growing body of evidence suggests that ecological speciation, defined as “the process by which barriers to gene flow evolve between populations as a result of ecologically based divergent selection between environments” [6], may be one of the key mechanisms of species formation in marine ecosystems (e.g. [7,34-37]). Population divergence is well known to occur along environmental gradients and may lead to the formation of new species [6,38]. Even weak selective gradients, as might occur with environmental gradients, can promote strong population divergence despite gene flow among continuously distributed populations [39]. Adaptation to local selective regimes can result in environment-phenotype mismatches such that larvae dispersing from their natal environment to a contrasting one would not survive to reproduce, effectively isolating populations [31,40]. Numerous theoretical and empirical studies suggest selection along environmental gradients (e.g. temperature, moisture, altitude, salinity) promotes adaptation to different suites of abiotic and biotic conditions and ultimately may impede gene flow, leading to speciation (reviewed in [6,41,42]).

While considerable evidence exists for each of these mechanisms influencing population structure of shallow-water organisms, evidence of them operating in the deep sea is limited, apart from hydrothermal vents and other chemosynthetic ecosystems that have been more intensively studied (reviewed in [43,44]). Several interesting patterns have begun to emerge from the few phylogeographic studies of deep-sea organisms in non-chemosynthetic environments. The most distinctive is that isolation by depth appears to be much greater than isolation by distance [45-50]. For example, population divergence based on mitochondrial markers was much greater for protobranch bivalves separated by 3km of depth than 10,000 km of geographic distance [48,51]. Another interesting pattern is that population divergence appears to decrease with depth, suggesting that continental margins might be the primary site of adaptive radiation for deep-sea organisms [47,52-54]. Probably the most surprising result to emerge is that population divergence can occur on extremely small scales despite the lack of obvious oceanographic or topographic features that might impede gene flow [55,56]. The small-scale divergence is often associated with depth differences and likely reflects the strong environmental gradients that attend changes in depth. In some cases the divergence is sufficient to be suggestive of cryptic species [57-60].

These emerging phylogeographic patterns suggest that the environmental gradients paralleling changes in depth likely play an important role in the formation of new species in deep-water ecosystems [47]. Increasing depth is associated with changes in a wide variety of environmental variables including temperature, hydrostatic pressure, oxygen, hydrodynamics, habitat heterogeneity, and the nature and amount of food [61]. Singly or in combination, these environmental changes are thought to influence the bathymetric distribution of organisms and shape many of the major macroecological patterns involving alpha and beta diversity [62-64]. While their potential ecological roles have long been appreciated, their influence on evolutionary processes and dynamics remains poorly understood.

Here we document patterns of connectivity and quantify the scale and geography of population divergence in a common protobranch bivalve *Nuculaatacellana* Schenck 1939 (formerly *Deminuculaatacellana* ) distributed across a depth gradient (1600-3800 m) in the western North Atlantic. Previous work using mtDNA (16S) identified strong genetic divergence among populations above and below 3000m [47,55]. These results were surprising because there were no obvious topographic or oceanographic features that might isolate populations from different depth regimes. Moreover, the distance separating these regions is less than 100 km, very likely within the dispersal window of *N* *. atacellana* ’s demersal pelagic larvae. Several explanations might account for the divergence including idiosyncrasies of mtDNA (e.g. smaller effective population size, gender-biased dispersal), selection due to environmental changes associated with depth, or the presence of bathymetrically separated cryptic species. To better evaluate these alternative explanations, we quantify phylogeographic patterns using five loci, including both mitochondrial and nuclear markers. Recent work has stressed the importance of using multiple loci because mutational and coalescent stochasticity can lead to incongruent patterns among independent loci (e.g. [65-69]), and phylogeographic patterns often differ between nuclear and mitochondrial loci (reviewed in [24,70]). Our results indicate that *Nuculaatacellana* has diverged across the depth gradient with very limited gene flow among bathyal and abyssal populations for more than 0.5 MY, possibly indicative of incipient speciation.

## Methods

### Ethics Statement

No specific permissions were required for collection of specimens, because they were collected in international waters below the continental shelf. Collection of specimens did not involve endangered or protected species.

### Cruise and specimen collection

**Figure 1:**
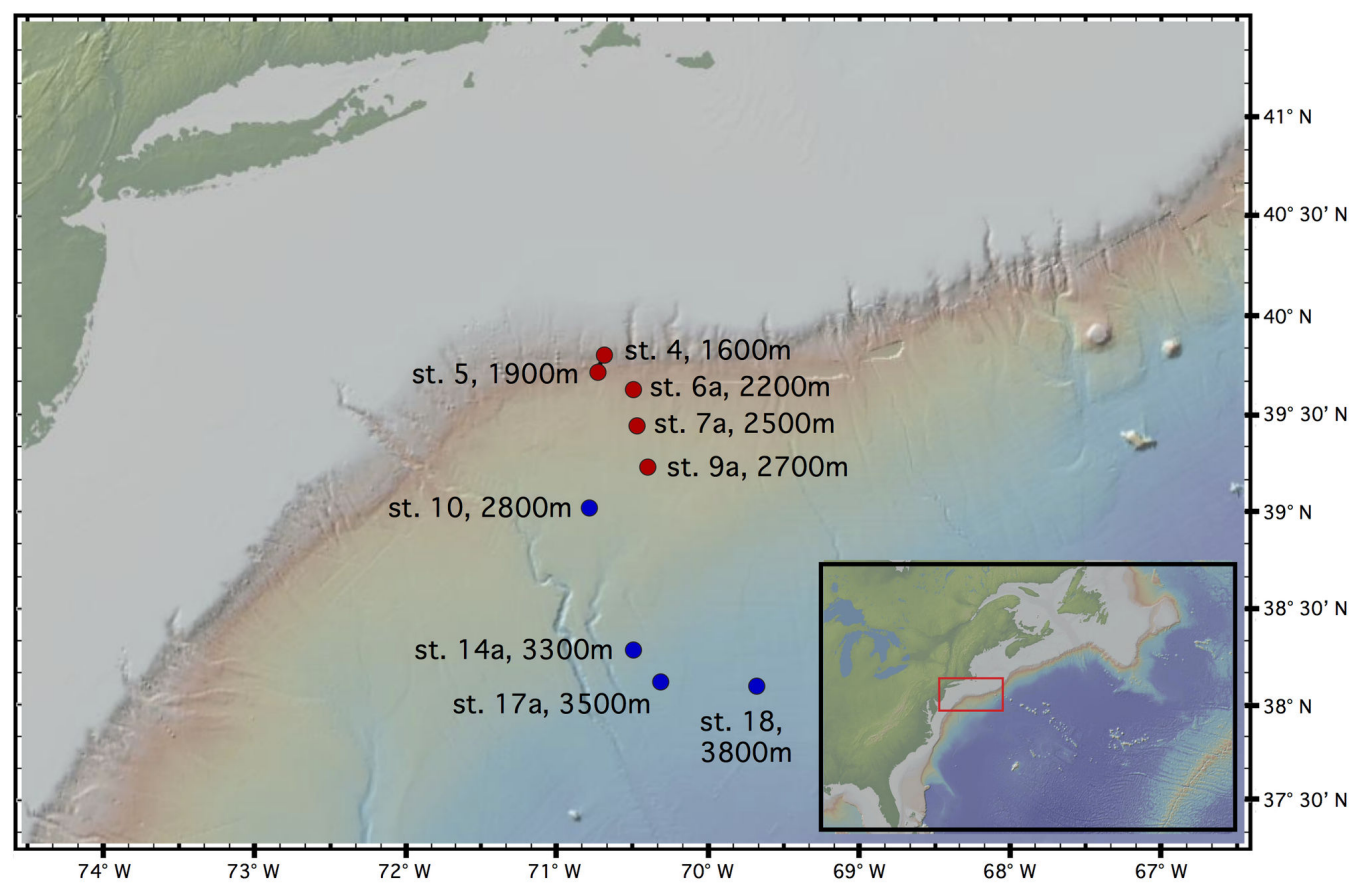
Map of sampled stations. The red box in the inset shows the location of the depth transect along the slope, rise, and abyssal plain of the Northwest Atlantic, with station names and depths indicated. Stations are color-coded according to a genetic separation between shallow (red) and deep (blue; see Results).

On a research cruise in 2008 on board the R/V *Endeavor*, specimens were collected along a transect closely following the Gay Head—Bermuda transect sampled by Hessler and Sanders [71]. At most stations two epibenthic sled tows were conducted; sediments were sieved and sorted live on board, in a chilled room (2°C) using chilled seawater to minimize stress to organisms. Following sorting, the remaining bulk samples were preserved in 95% ethanol and kept at –20°C. *Nuculaatacellana* specimens sorted on board were either preserved by flash-freezing or by placing in 95% ethanol, and stored at –80°C. Additional specimens were sorted from the bulk samples after the cruise, also using chilled ethanol to slow DNA degradation. *N* *. atacellana* was collected at 9 of 20 stations (Figure 1, Table 1), across a depth range of 1600–3800m.

**Table 1:** Sampled station information, with coordinates, depths, and N collected.

Station	Depth (m)	Lat (°N)	Lon (°W)	N	
4	1600	39.7807	70.7091	14	
5	1900	39.7593	70.7132	12	
6a	2200	39.6367	70.5033	30	
7a	2500	39.4500	70.4667	12	
9a	2700	39.2403	70.3993	8	
Shallow Group				76	
10	2800	39.0371	70.7812	6	
14a	3300	38.2952	70.4940	2	
17a	3500	38.1333	70.3167	5	
18a	3800	38.1050	69.6933	6	
Deep Group				19	
Total				95	

### DNA extraction and locus amplification

Genomic DNA was extracted using the QIAamp Mini DNA Extraction Kit (Qiagen, Valencia, CA), using the standard animal tissue protocol with 2 sequential elutions of 100µL. PCR amplifications of mitochondrial COI and four noncoding nuclear loci (an actin intron (MAC), a calmodulin intron (CAL), and two noncoding anonymous fragments (DAC3 and DAC6)) were performed separately. Anonymous DNA markers were obtained by digestion of genomic DNA with the restriction enzyme AluI (NEB, Ipswich, MA), agarose gel selection of 1.0-1.5 kb fragments, cloning and sequencing; candidate markers were screened for polymorphism by sequencing a subset of the above specimens. Nuclear introns were selected based on a previous survey of introns in protobranch bivalves [72]. Standard PCR reaction mixtures were employed, and thermocycler conditions optimized for each locus (Table S1). In the few cases of poor amplification under these conditions, reamplification was performed with both a new negative control and reamplification of the original negative control, using nested primers where possible.

### Sequencing, Heterozygote Detection, and Alignment

All successful amplifications produced single PCR bands as seen by gel electrophoresis, except for some individuals heterozygous at CAL for a 68bp indel that allowed separation of the two alleles in the gel. These alleles were gel purified and sequenced separately; all other single PCR bands were sequenced regardless of heterozygous status. Bi-directional sequencing was performed by Agencourt (a Beckman-Coulter company, Beverly, MA). The two reads for each individual were trimmed, aligned, and manually edited using Sequencher 5.0.1 (Gene Codes Corp., Ann Arbor, MI).

Individual base pairs were considered heterozygous if a clear double peak of near-equal height existed in both chromatograms, in the context of otherwise low or nonexistent background. Heterozygotes possessing alleles of different lengths (polymorphic indels) were ascertained by initially clear, single-peaked chromatograms that became almost-totally double-peaked (except for runs of a single nucleotide) while maintaining well-shaped peaks and regular spacing. The two juxtaposed sequences were deconvolved with the online program Indelligent [73]; each strand was deconvolved separately and the estimated alleles realigned to each other for editing and quality control. Remaining heterozygous positions were phased using PHASE 2.1.1 [74,75], employing the Parent-Independent Mutation (PIM) model for sites containing indels or more than two bases. Any uncertain phases were estimated with a second run using haplotypes phased with certainty 1.00 as knowns (–k option). Sequences were aligned using the CLUSTAL algorithm [76] in BioEdit with default alignment parameters. For nuclear loci, both alleles of all individuals were included in the alignment. Final alignments were trimmed and checked manually.

### Basic and Within-Locus Analyses

To ensure the noncoding status of nuclear loci, they were checked for the potential presence of coding sequences by BLAST searches against the GenBank nucleotide database, GenBank’s ORF finder, the Gene Ontology database BLAST2GO [77], and AUGUSTUS [78]. Potential RNA secondary structure formation was assessed with Mfold [79].

Arlequin 3.5 [80] was used to compute basic indices and statistics for each locus separately: the number of haplotypes (Nhap), haplotypic diversity (H), and nucleotide diversity (π). Tajima’s D (tested at α=0.05), and Fu’s Fs (tested at α=0.02) were computed as basic tests of neutrality and demographic stability. Note that Arlequin excludes gapped positions (e.g. indels) when determining haplotypes; therefore, haplotype counts and diversity differ from other estimates. Based on initial indications of strong genetic separation at COI between a shallow group (stations 4–9a, 1600–2700m) and a deep group (stations 10–18/18a, 2800–3800m), indices and statistics were computed for each individual station with n≥3, for stations pooled among the shallow and deep group, and for all pooled individuals. For nuclear loci, deviations from Hardy-Weinberg equilibrium (HWE) were determined in Arlequin using default settings, computed among whole haplotypes. Within each locus, estimated recombination rates were determined between successive base pairs in PHASE [81,82], assuming a threshold of >5x background. Linkage disequilibrium (LD) among the nuclear loci was tested in Arlequin, using 1000 dememorization steps and 1,000,000 steps in the Markov chain.

As an additional test of selection, the McDonald-Kreitman test was performed by hand on COI, between the shallow and deep groups. Statistical significance was determined by computing the χ^2^ statistic and p-value for the 2-by-2 contingency table of differences: Fixed vs. Variable and Synonymous vs. Nonsynonymous [83].

### Population Clustering Analyses

Structure v2.3.4 [67] was used to determine the most likely number of populations (K) and to assign individuals to putative populations. The admixture model was employed, estimating separate α’ s for each population, and setting λ=1 (the Dirichlet parameter for allele frequencies) for all populations. Allele frequencies among putative populations were modeled as uncorrelated (discussed in 84), and the chain was run with a burn-in of 100,000 steps followed by 500,000 steps. Twenty replicate runs per K were conducted for K=1 to K=10, and Structure Harvester [85] was used to choose K using the delta K criterion [86]. Because K=1 cannot be evaluated using delta K, the method of Pritchard et al. [67] for choosing K was also calculated. Output for the chosen K was analyzed in CLUMPP [87] using greedy heuristic searches with 5000 random permutations, and resulting admixture proportions were plotted using *distruct* [88].

Haplotype networks were constructed for individual loci in TCS [89], treating gaps as a 5^th^ base (except for COI, where they represented missing data) and increasing the connection limit until all haplotypes were incorporated into a single network. For CAL, a large 68-bp indel (see Results) required two networks, one for the four “deletion haplotypes” and one for the remaining “insertion haplotypes”.

The population clustering determined by Structure (see Results) was tested in Arlequin by AMOVA on all five loci, nesting individuals within stations, and stations within the shallow and deep groups. AMOVAs on multilocus and locus-by-locus pairwise differences were calculated with significance assessed from a null distribution of 1000 randomizations; multilocus pairwise Φ_ST_ and Φ’_ST_ values (Φ_ST_ standardized by its maximum attainable value [90]) were also calculated and tested for significance within this AMOVA framework. To test for isolation-by-distance (IBD) within each population, we regressed Slatkin’s linearized Φ_ST_ against pairwise measures of (1) the log of geographic great-circle distance between stations and (2) the log of depth difference between stations, separately within the shallow and deep groups identified by Structure. Regression was performed via partial Mantel tests in Arlequin [91], removing the effect of depth on distance, and of distance on depth. Significance was assessed from a null distribution of 1000 random permutations.

### Demographic and Population Genetic Analyses

The demographic history of populations was reconstructed in IM v.12.17.09 [92], using the populations determined by Structure and verified by AMOVA. The HKY mutation model was chosen for all loci, with a mutation rate for COI of 0.45%/(lineage·site·million-years), taken from an analysis of arcid bivalves by Marko [26]; mutation rates for nuclear loci were not specified. An Exponential Population Size Change Model was used because Extended Bayesian Skyline Plots (EBSP; see below and Results) indicated an exponentially growing shallow population. Separate analyses were conducted with and without COI. When COI was excluded, a mutation rate for CAL was used that had been estimated in BEAST calibrated with COI. Initial runs of 50,000 burn-in followed by 100,000 steps were performed to determine proper upper bounds for priors on population size (q), splitting time (t), and migration rates (m). Longer runs (>10^8^) employed 10 Metropolis-coupled chains with a two-step increment model (as per the manual) and a burnin of 100,000, and were continued until all ESSs > 70. Three replicate runs with different starting seeds were performed to assess convergence. Parameters were converted to “demographic units” using a heuristic generation time of 10 years. The inferred demographic history was plotted using IMfig. An Extended Bayesian Skyline Plot (EBSP; [93]) was produced in BEAST 1.7.4 [94] as further analysis of demographic history. Convergence was assessed using Tracer 1.5, and demography plotted using scripts written by J. Heled (https://code.google.com/p/beast-mcmc/downloads/detail?name=EBSP.zip&can=2&q=).

To determine the evolutionary history of populations and individuals, two applications of starBEAST [95] were used. In both applications, only the subset of fully sequenced individuals was used (n=74). Separate partitions were created for COI (single haplotype per individual) and each of the nuclear loci (both alleles included for all individuals), with substitution models, clock models, and locus trees unlinked across loci. The “SRD06” mutation model was used for COI, and each nuclear locus was given a GTR model with estimated equilibrium nucleotide frequencies and four categories of gamma-distributed rate variation. All loci were modeled with uncorrelated lognormally distributed clocks, setting the mean COI rate to 1.0 and estimating the others relative to COI. Starting trees were obtained by UPGMA, and a Yule prior was enforced with a piecewise linear population size and a constant root. Default priors were used for all parameters except for relative mutation rate priors for COI and clock mean rate priors, which were set to normal distributions with means and standard deviations of 1. Operators were tuned automatically, with weights adjusted per the BEAST manual. The MCMC chain was run for 10^7^ steps; burnin was determined with Tracer 1.5 and consensus trees obtained with TreeAnnotator 1.7.4. In the first application, a “population tree” was created by assigning each individual to the population inferred with Structure. In the second application, a genealogy of individuals was created by assigning all nine sequences of an individual to that individual.

## Results

### Within-Locus Indices and Tests

Four nuclear loci and one mitochodrial locus were successfully sequenced from 95 individuals collected from 9 stations along a depth gradient from 1600-3800 m in the western North Atlantic (Table 2). Heterozygous indels were detected in all four nuclear loci: the 68bp indel in CAL was flanked by two indels of 4bp each, and the MAC intron contained six 1bp indels, one 2bp indel, and one 5bp indel. DAC3 contained a short run of TA repeats, and DAC6 contained 4 short indels (1bp, 1bp, 3bp, and 5bp). All sequences were deposited in GenBank (Accessions KC563091-KC563901, Table 2). No significant BLAST matches were found in the four nuclear loci, nor were ORFs or likely RNA secondary structures detected. Among all loci, DAC3 had the most haplotypes, followed by MAC, COI, CAL, and DAC6. Haplotype diversity was consistently very high; however, the deep group showed noticeably lower haplotype diversity at COI (Two-way ANOVA with locus and depth group as factors, p<0.001; Tukey’s post-hoc comparison of deep COI vs. shallow COI p<0.001). Tests of Hardy-Weinberg equilibrium showed no departures from neutral expectations, and tests of LD showed no disequilibrium among the nuclear loci (Table 3). For recombination, one location (bp 11–12 in MAC) showed evidence of a recombination rate 11x above background, but variance in this estimation within the PHASE run was greater than the mean.

**Table 2:** Alignment length, basic statistics, and neutrality indices.

**LOCUS: length (bp)**	**Station**	**N seq**	**Nhap**	**H**	**π**	**Tajima’s D**	**Fu’s Fs**
COI: 651	4	14	14	1.0000	0.0391	**-1.8636**	-3.0226
	5	12	12	1.0000	0.0390	**-1.8096**	-2.1712
	6a	30	28	0.9931	0.0369	**-1.9209**	**-8.2027**
	7a	11	11	1.0000	0.0430	-1.1550	-1.5718
	9a	7	7	1.0000	0.1404	-1.0090	1.3799
	Shallow Group	74	66	0.9930	0.0483	**-2.2523**	**-24.0637**
	10	6	3	0.6000	0.0077	**-1.4725**	2.9600
	14a	2	1	NC	NC	NC	NC
	17a	5	2	0.4000	0.0006	-0.8165	0.0902
	18 and 18a	6	2	0.3333	0.0005	-0.9330	-0.0028
	Deep Group	19	3	0.5088	0.0065	0.4950	6.4760
	Total	93	69	0.9759	0.0537	-0.9854	**-15.8231**
GenBank Accessions:	KC563091-KC563183						
CAL: 213	4	14	12	0.8810	0.1637	-0.6471	9.9960
	5	12	14	0.9457	0.0937	-1.3462	1.8912
	6a	30	27	0.9328	0.1568	**-2.0539**	4.3031
	7a	12	20	0.9819	0.1155	-0.5346	-2.0350
	9a	7	8	0.9011	0.1598	-0.9671	6.3583
	Shallow Group	75	57	0.9389	0.1396	-1.3957	-0.7906
	10	6	8	0.9394	0.0162	-0.4816	-2.1856
	14a	2	4	NC	NC	NC	NC
	17a	5	7	0.9111	0.1715	-0.9910	4.2845
	18 and 18a	5	8	0.9556	0.0887	0.5574	1.0135
	Deep Group	18	17	0.9508	0.0883	-0.3933	2.3883
	Total	93	69	0.9484	0.1322	-1.397	-3.5017
GenBank Accessions:	KC563184-KC563369						
MAC: 254	4	12	15	0.9203	0.0464	1.5091	-0.8412
	5	12	5	0.6957	0.0480	1.9469	11.0681
	6a	29	25	0.8814	0.0464	1.3970	-1.6835
	7a	11	17	0.9784	0.0560	0.7653	-2.2385
	9a	8	13	0.9750	0.0672	1.2362	-0.9699
	Shallow Group	72	62	0.9079	0.0508	1.0397	**-20.1198**
	10	3	5	0.9333	0.0220	-0.1057	-0.2168
	14a	2	3	NC	NC	NC	NC
	17a	2	4	1.0000	0.0291	0.2791	-0.0653
	18 and 18a	5	8	0.9556	0.0229	1.0291	-1.6752
	Deep Group	12	20	0.9819	0.0195	0.2111	**-7.3103**
	Total	84	77	0.9315	0.0485	0.8064	**-24.0620**
GenBank Accessions:	KC563370-KC563537						
DAC3: 296	4	14	17	0.9286	0.0141	**-1.7403**	**-7.5118**
	5	12	15	0.8659	0.0102	-1.4193	**-8.5114**
	6a	30	25	0.9249	0.0136	**-1.8374**	**-11.6838**
	7a	12	20	0.9855	0.0187	-0.9744	**-12.4493**
	9a	6	9	0.9545	0.0219	-1.3440	-1.4768
	Shallow Group	74	66	0.9471	0.0221	**-2.2682**	**-25.5301**
	10	6	9	0.9545	0.0114	0.5887	**-3.6440**
	14a	2	3	NC	NC	NC	NC
	17a	4	6	0.9286	0.0114	-0.5409	-1.3732
	18 and 18a	5	6	0.8889	0.0078	0.3845	-1.5081
	Deep Group	17	17	0.9055	0.0095	-0.4450	**-9.4151**
	Total	91	81	0.9495	0.0154	**-2.2212**	**-25.4005**
GenBank Accessions:	KC563538-KC563719						
DAC6: 333	4	14	10	0.8942	0.0513	0.8095	6.8154
	5	12	6	0.7826	0.0329	1.2646	8.2304
	6a	27	17	0.8917	0.0443	0.2571	3.7999
	7a	12	14	0.9239	0.0421	-0.3096	0.5763
	9a	8	10	0.9333	0.0220	-0.6046	-0.6332
	Shallow Group	73	33	0.9207	0.0416	0.2287	0.5957
	10	6	8	0.9091	0.0074	0.1794	**-3.295**
	14a	2	2	NC	NC	NC	NC
	17a	4	4	0.7857	0.0074	1.1762	0.5530
	18 and 18a	6	5	0.8485	0.0085	1.5085	0.5711
	Deep Group	18	11	0.8571	0.0087	-0.0554	-1.9937
	Total	91	38	0.9025	0.0368	-0.0060	-1.1553
GenBank Accessions:	KC563720-KC563901						

Nseq, number of individuals sequenced; Nhap, number of haplotypes detected; H, haplotype diversity; π, nucleotide diversity. Tajima’s D values are bolded if significant at α=0.05 and Fu’s Fs if significant at α=0.02.

**Table 3:** A, Test of Hardy-Weinberg equilibrium (HWE) using whole haplotypes at all loci; B, P-values for tests of linkage disequilibrium using whole haplotypes at the four nuclear loci.

A.					
	Sample	p-value			
	st. 4	0.7136			
	st. 5	1.0000			
	st. 6a	1.0000			
	st. 7a	0.7941			
	st. 9a	1.0000			
	Shallow group	0.9944			
	st. 10	1.0000			
	st. 14a	1.0000			
	st. 17a	1.0000			
	st. 18	0.1591			
	Deep group	0.8928			
**B.**					
		CAL	MAC	DAC3	DAC6
	CAL	–	0.1660	0.4850	0.2790
	MAC		–	0.5110	0.0550
	DAC3			–	0.2990
	DAC6				–

Simple tests of neutrality revealed a few significantly negative values for Tajima’s D at several loci (Table 2), with departures from neutrality more common and negative at COI and DAC3, less so at MAC and CAL, and not detected at DAC6. The McDonald-Kreitman test for COI revealed a ratio of polymorphic nonsynonymous to synonymous sites (Pn/Ps) of 0.0482, and a ratio of fixed nonsynonymous to synonymous sites (Dn/Ds) of 0.0625; Pn/Ps< Dn/Ds implies that potential selection is negative. The Neutrality Index was 0.771 (NI, calculated as (Pn/Ps)/(Dn/Ds)), corresponding to a proportion of selected sites, α, of 1-NI=0.229. The 2-by-2 contingency table χ^2^ statistic was 0.0513 (p=0.821), indicating that COI is not under selection.

### Population Clustering Analyses

**Figure 2:**
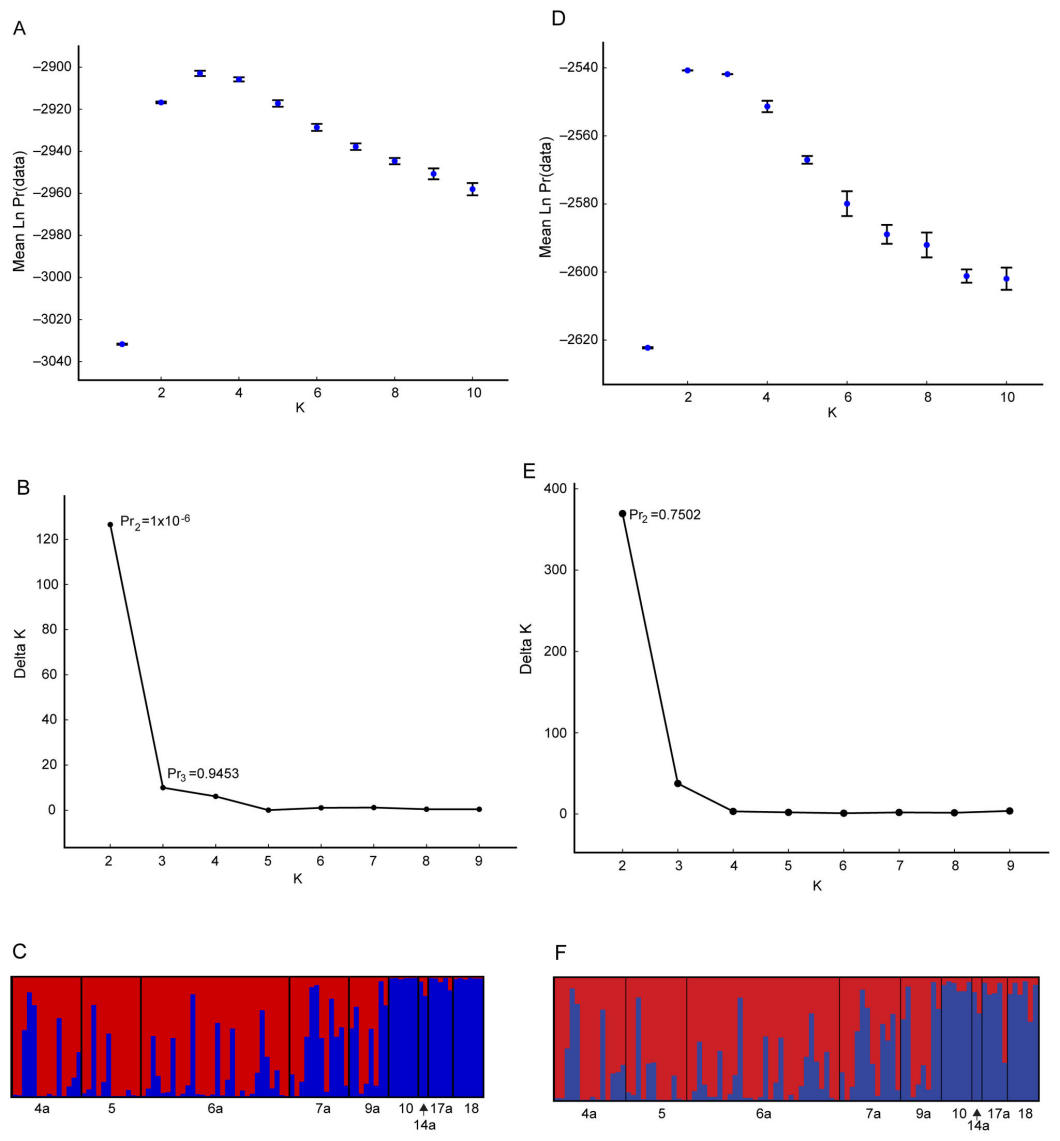
Structure analysis for all loci and just nuclear loci. A, Mean likelihood scores and standard deviations from 20 replicate runs at each K from Structure analysis of all 5 loci. B, Plot of Delta K model scores using the Evanno et al. method (2005); “Pr” indicates the probability for the best model (K=3) according to the method of Pritchard et al. (2000) and for the Evanno-selected K=2. C, Admixture proportions for the most likely grouping (K=2). D–F, the same analyses and measures for Structure analysis on nuclear loci only.

Structure runs tended to exhibit small variance at the highest and lowest Ks (K≤3 and K≥8), with larger variance at intermediate K (Figure 2A); however, significantly lower likelihood scores at intermediate Ks resulted in the clear choice of K=2 based on the Evanno criterion (Figure 2B); applying the Pritchard criterion resulted in K=3, with support for no separation (K=1) essentially zero. Although admixture proportions were relatively variable within the shallow group, shallow vs. deep individuals were generally ascribed to separate groups (red vs. blue respectively; Figure 2C). Structure analysis of just the nuclear loci produced slightly different admixture proportions, but resulted in a clear choice of K=2 by Evanno and Pritchard criteria (Figure 2 D-F), and again essentially zero support for K=1. Assignment proportions for this K=2 configuration indicated that 76 individuals belonged to the shallow group, and 19 to the deep group.

The differential admixture of individuals at mitochondrial vs. nuclear loci was apparent in haplotype networks (Figure S1 vs. S2-S5). While all networks showed high allelic diversity, haplotypes of deep individuals were separated more in COI than in nuclear networks.

**Figure 3:**
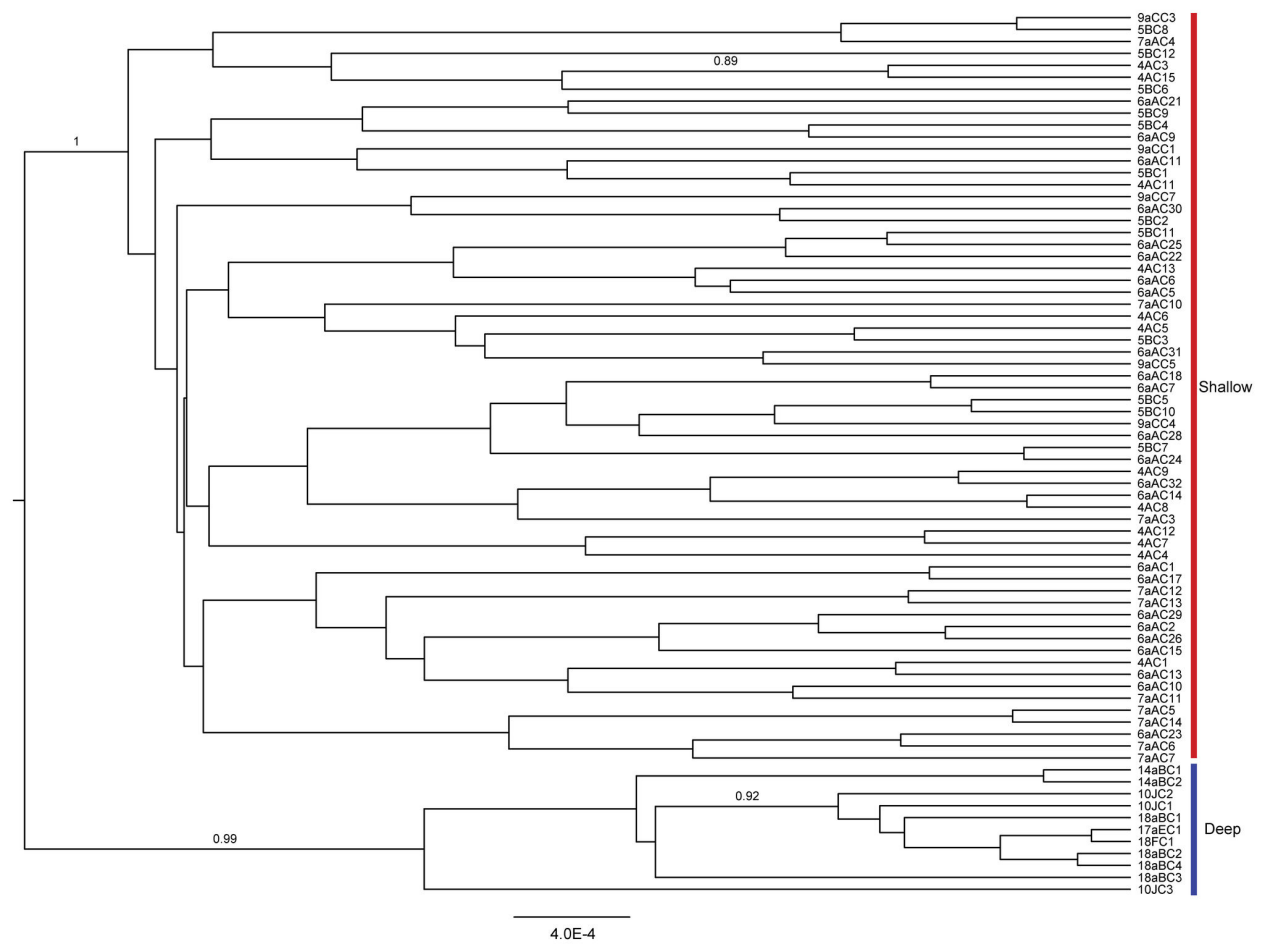
The starBEAST genealogy. Branch lengths are proportional to substitutions per site, combined across loci. Bayesian posterior clade probabilities are shown if >0.75, and population assignments are colored as in Figure 1.

The AMOVA confirmed the Structure (K=2) results, indicating significant divergence between shallow and deep populations for each locus independently and when all loci were analyzed together, with little divergence within populations (Table 4). The congruence between nuclear loci and COI indicated that the depth-related divergence occurred across all loci and was not exclusive to the mitochondrion. Across all five loci, Φ_ST_’ s were generally higher between shallow and deep pairs than among shallow pairs or among deep pairs (Table 5); the standardized Φ’_ST_ had the same pattern of significant pairwise values (not shown). Particularly among deep stations, significant Φ_ST_’ s likely reflect small sample sizes. The significant separation of shallow and deep lineages was highly supported in the starBEAST genealogy (posterior probability 0.99-1.00, Figure 3), with no nodal support for significant substructure within either group. Although strong divergence was detected between depth regimes, we found little spatial structure within (Table 5). Isolation by depth was not detected within the shallow or deep populations; isolation by distance was statistically significant in the shallow group, but did not remain significant when the effect of depth was removed (Table 6).

**Table 4:** AMOVA analyses within each locus and with all loci combined.

Source of Variation	Locus	d.f.	Sum of Squares	Variance Components	Percentage Variation	p-value
Among Groups	All	1	961.671	14.7033	27.84	**<0.001**
	Nuclear	1	136.104	1.7928	8.00	**0.0049**
	COI	1	422.688	13.4951	50.76	**0.0108**
	CAL	1	66.294	0.8490	5.79	**0.0411**
	MAC	1	30.801	0.4997	7.61	**0.0059**
	DAC3	1	34.748	0.5635	21.01	**0.0049**
	DAC6	1	51.847	0.7614	11.53	**0.0098**
Among Populations,	All	7	484.910	0.3851	0.73	**<0.001**
Within Groups	Nuclear	7	185.100	-0.1622	-0.72	0.3275
	COI	7	105.061	0.2177	0.82	**0.0362**
	CAL	7	116.034	-0.0996	-0.68	0.4379
	MAC	7	74.205	0.0546	0.83	0.1075
	DAC3	7	27.019	0.0638	2.38	**0.0078**
	DAC6	7	49.323	-0.1866	-2.83	0.5298
Among Individuals,	All	86	5289.156	23.7825	45.04	**0.0327**
Within Populations	Nuclear	86	2555.643	8.9452	39.93	**<0.001**
	COI	84	1081.455	12.8745	48.42	**<0.001**
	CAL	84	1557.043	4.6176	31.48	**<0.001**
	MAC	75	722.232	3.6214	55.18	**<0.001**
	DAC3	82	216.315	0.5827	21.72	**<0.001**
	DAC6	82	876.825	4.6624	70.59	**<0.001**
Within Individuals	All	95	1324.000	13.9368	26.39	**<0.001**
	Nuclear	95	1123.500	11.8263	52.79	**<0.001**
	COI	–	–	–	–	–
	CAL	93	865.000	9.3011	63.41	**<0.001**
	MAC	84	200.500	2.3869	36.37	**<0.001**
	DAC3	91	134.000	1.4725	54.89	**<0.001**
	DAC6	91	124.500	1.3681	20.71	**<0.001**
Total	All	189	8059.737	52.8077		
	Nuclear	189	4000.347	22.4021		
	COI	92	1609.204	26.5873		
	CAL	185	2604.371	14.6681		
	MAC	167	1027.738	6.5626		
	DAC3	181	412.082	2.6825		
	DAC6	181	1102.495	6.6053		

d.f., degrees of freedom. P-values are bolded if significant at α=0.05.

**Table 5:** Pairwise Φ_ST_ values among sampled populations.

A. Pairwise Φ_ST_, all loci									
	st4	st5	st6a	st7a	st9a	st10	st14a	st17a	st18
st4	–								
st5	0.0284	–							
st6a	-0.0079	0.0307	–						
st7a	0.0319	0.0438	0.0216	–					
st9a	0.0808	0.1048	**0.0882**	0.0756	–				
st10	**0.3497**	**0.3620**	**0.3295**	**0.3239**	**0.3469**	–			
st14a	0.3394	**0.3784**	**0.3438**	**0.3259**	0.3132	**0.4807**	–		
st17a	**0.2524**	**0.3139**	**0.2593**	**0.2592**	**0.2471**	**0.1738**	0.4075	–	
st18	**0.3233**	**0.3705**	**0.3135**	**0.3243**	**0.3375**	**0.0807**	**0.5128**	-0.0233	–
B. Pairwise Φ_ST_, nuclear loci									
	st4	st5	st6a	st7a	st9a	st10	st14a	st17a	st18
st4	–								
st5	0.0507	–							
st6a	-0.0164	0.0497	–						
st7a	0.0089	0.0009	0.0025	–					
st9a	-0.0022	0.0475	0.0013	0.0162	–				
st10	**0.1987**	0.1137	**0.1783**	**0.1300**	0.2232	–			
st14a	0.1522	0.1203	0.1449	0.0927	0.1938	**0.2352**	–		
st17a	-0.0112	0.0394	0.0105	0.0146	-0.0624	**0.1755**	0.1610	–	
st18	0.0891	0.0617	0.0797	0.0538	0.0737	**0.1027**	0.1577	-0.0318	–

Statistically significant values are in bold. Station numbers are listed by increasing depth with a line separating shallow stations (4–9a) from deep stations (10a–18).

**Table 6:** Mantel and partial Mantel tests of isolation-by-distance and -by-depth.

		Slatkin’s Linearized Φst	
		r	p
Shallow Group	Distance	0.578	0.031
	Depth	0.334	0.141
	Distance (depth removed)	0.501	0.116
	Depth (distance removed)	-0.035	0.424
Deep Group	Distance	-0.320	0.795
	Depth	-0.229	0.729
	Distance (depth removed)	-0.429	0.781
	Depth (distance removed)	0.372	0.363

All spatial variables were converted to log(km).

### Demographic History

**Figure 4:**
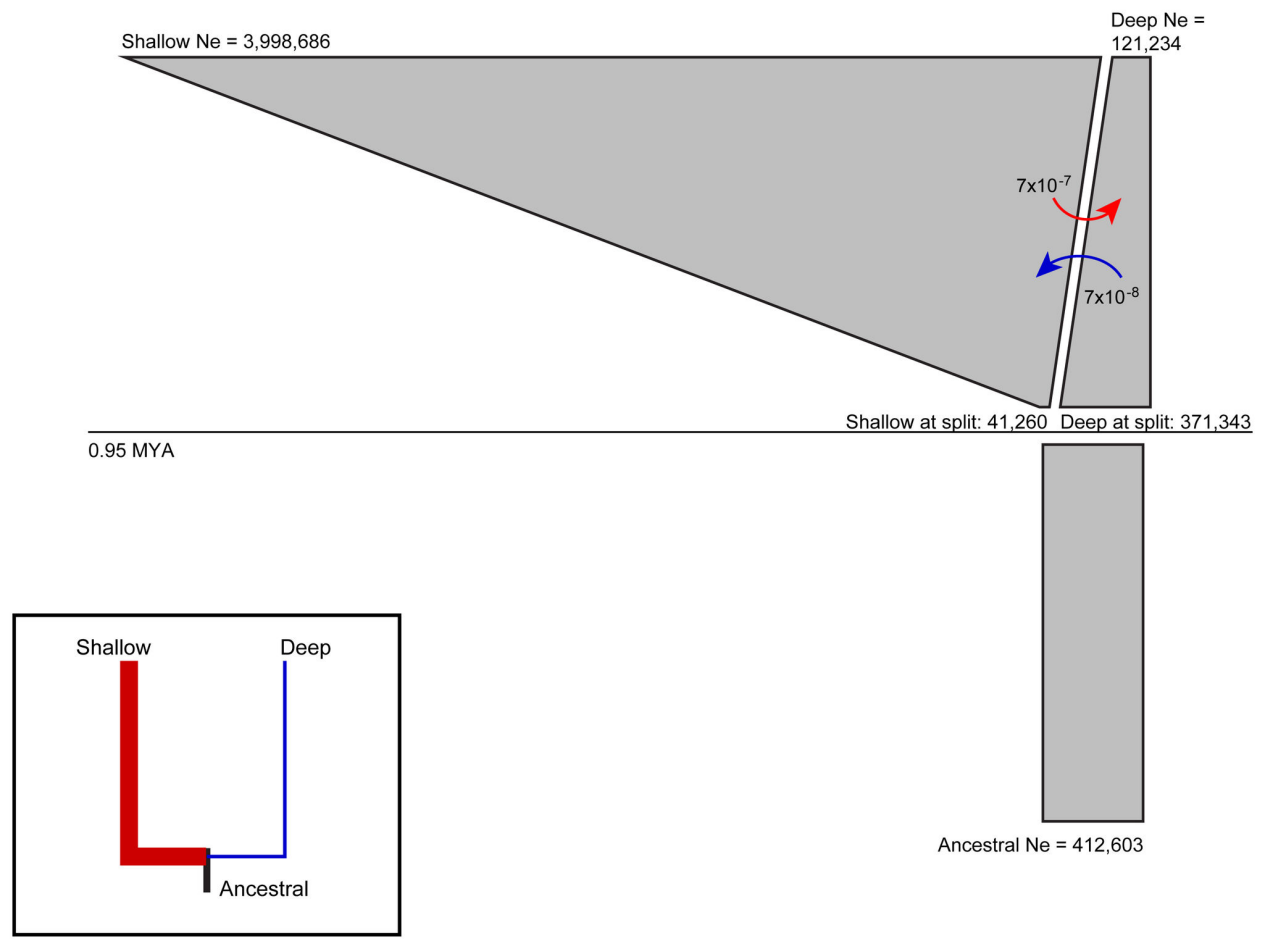
Population demographic history and migration estimates from IM for all loci. The gray box indicates the estimated effective population size (Ne) of the ancestral population. Estimated splitting time is indicated by the horizontal line. Descendant shallow and deep populations are represented above the line by polygons whose starting width is the estimated Ne just after the split and whose upper width is the estimated contemporary Ne. Curved dotted arrows represent estimated migration rates per generation, forward in time from source to destination. Demographic history estimation from starBEAST is shown in the inset, with branch thickness proportional to estimated population size. Coloring of shallow and deep is as in Figure 1.

Coalescent reconstruction of demographic history as estimated in IM using all 5 loci revealed an ancestral population that split approximately 95,000 generations in the past, resulting in largely independent shallow and deep populations along the sampled depth gradient (Table 7A, Figure 4). Although no good estimate exists for protobranch generation times, a conservative value of 10 years (an estimate within the range reported by Zardus [96]) translates to a split of 0.95 million years ago (MYA). The demographic estimates indicated an ancestral effective population size of ~412,000, a smaller deep population (N_e_~121,000), and a much larger shallow population (N_e_~3,998,000), comparable to the relative population sizes produced by the starBEAST population analysis (Figure 4, inset). Migration rates between populations per generation were extremely low (10^-7^ -10^-8^) and asymmetric with greater dispersal from the shallow to the deep population. Translation of these estimates into demographic units indicates that, of the 4 million individuals in the shallow population, approximately 5 migrate to the deep population each generation. Results were qualitatively similar for the IM analyses excluding COI (Table 7B). Effective populations sizes were lower, the splitting time was more recent and migration rates were somewhat larger (10^-6^ -10^-7^), with overall migration still extremely low. All three replicate IM runs for each analysis (with and without COI) produced very similar estimates with 95% HPD overlapping extensively for all parameters (not shown).

**Table 7:** Demographic and historical parameter estimates from IM.

**A. All loci**					
**Population Size**	**Theta**	**95% HPD**		**Ne (x1000)**	**95% HPD**
Shallow	184.83	(109.01, 346.35)		3998.686	(2358.461, 7439.080)
Deep	5.60	(3.34, 9.09)		121.234	(72.333, 196.623)
Ancestral	19.07	(13.51, 26.61)		412.603	(292.388, 575.607)
**Migration Rates**	**m**	**95% HPD**		**Migration Rate**	**95% HPD**
Deep to Shallow	0.6370	(0.189, 1.389)		7x10^-8^	(1x10^-8^, 1x10^-7^)
Shallow to Deep	0.0585	(0.011, 0.116)		7x10^-7^	(2x10^-7^, 2x10^-6^)
**Splitting Time**	**Tau**			**Years (Millions)**	
	1.101	(0.759, 1.575)		0.953	(0.657, 1.363)
**B. Nuclear loci**					
**Population Size**	**Theta**	**95% HPD**		**Ne (x1000)**	**95% HPD**
Shallow	49.76	(37.73, 66.43)		318.319	(241.330, 424.919)
Deep	6.34	(3.66, 11.16)		40.567	(23.393, 71,363)
Ancestral	10.51	(6.90, 19.77)		67.217	(44.120, 126.439)
**Migration Rates**	**m**	**95% HPD**		**Migration Rate**	**95% HPD**
Deep to Shallow	0.7525	(0.278, 1.433)		3x10^-7^	(1x10^-7^, 6x10^-7^)
Shallow to Deep	0.0735	(0.029, 0.155)		3x10^-6^	(1x10^-6^, 6x10^-6^)
**Splitting Time**	**Tau**			**Years (Millions)**	
	2.186	(1.706, 3.602)		0.559	(0.438, 0.922)

The 95% highest posterior density (HPD) is given in parentheses. Ne, effective population size; Migration Rate, estimated migration rate per generation, forward in time from source to destination.

**Figure 5:**
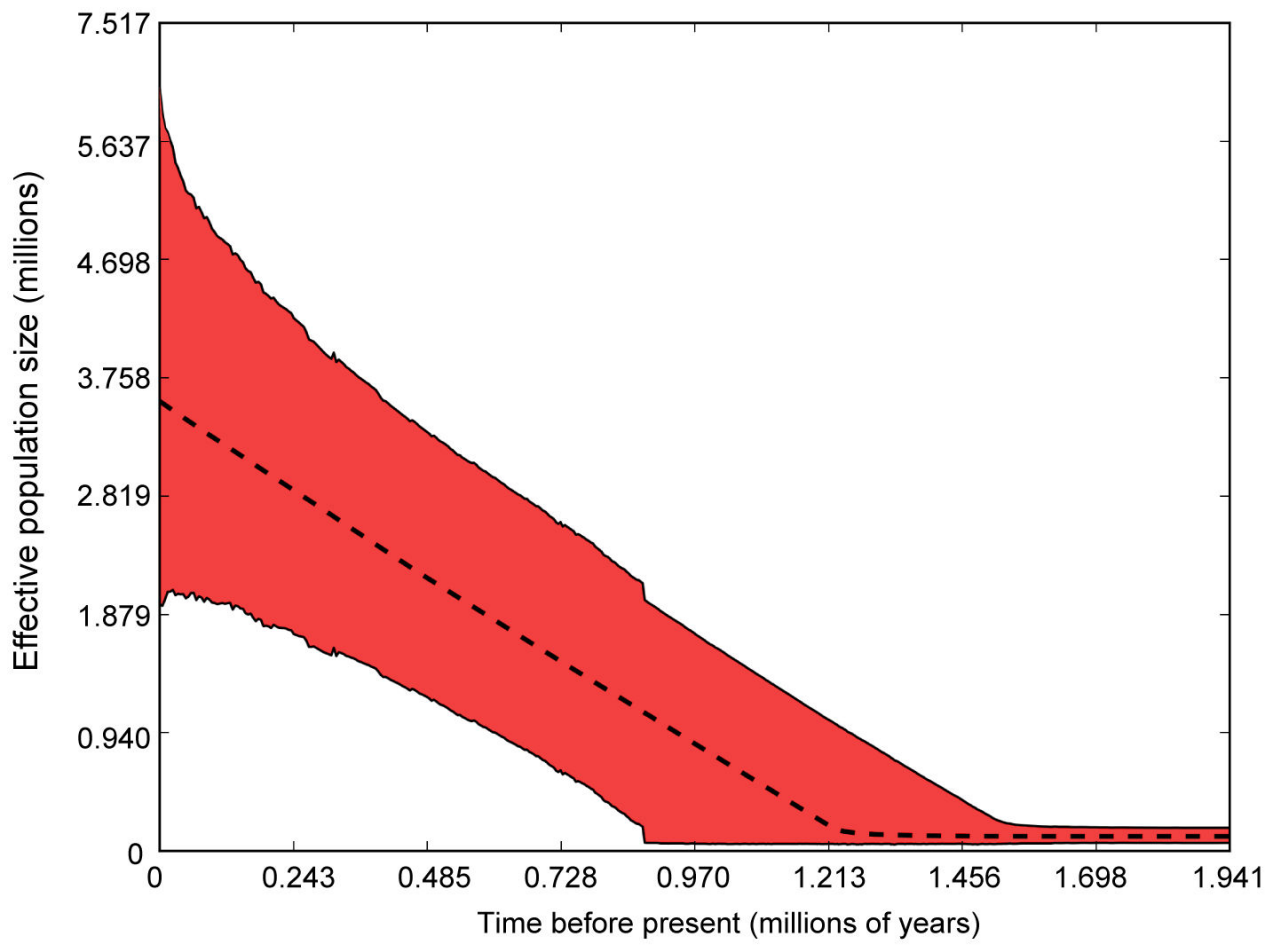
Extended Bayesian Skyline Plot of population size through time for the shallow population. Median (line) and 95% HPD (red shading) population size are shown.

The EBSP analysis of shallow population history also showed a likely increase in the shallow population size from its ancestral size to a current N_e_ of 2 to 3 million, over approximately the last 1MY (Figure 5). Median population size of the deep group EBSP indicated population growth starting about 0.023 MYA (not shown), but the 95% highest posterior distribution (HPD) was quite large, including both zero growth and unrealistically high growth. The smaller sample size of the deep population appears to increase the error around demographic reconstruction.

## Discussion

### A strong genetic break across a depth gradient

The most striking feature of the phylogeographic analysis of *N* *. atacellana* is a sharp genetic break at 2700m across just 100m depth and 40km horizontal distance. Although the divergence is most obvious for the mitochondrial locus with no shared haplotypes between shallow and deep populations (Figure S1), the nuclear loci all show significant population structure (Tables 4 and 5, Figures S2–S5) and all analytical results were qualitatively the same when COI was excluded. The location of the break is quite similar to previous findings for *N* *. atacellana* [47,55], but more intense sampling narrowed the depth separation between shallow and deep populations to 100m. Within depth regimes (i.e. 1600-2700m, and 2800-3800m), very little genetic differentiation was detected using Structure, AMOVA, or a Mantel test for IBD. Multilocus coalescent modeling suggests that the depth divergence reflects a historical population split some 0.95 MYA, with extremely low gene flow across the break since its inception. When COI was excluded, Structure, AMOVA, and IM still detected divergence between shallow and deep populations; although the estimated splitting time was younger (0.55 MYA; Figure S6). Taken together, therefore, there is strong evidence from both nuclear and mitochondrial loci for a significant genetic break between populations in close proximity along the depth gradient, and little divergence within shallow and deep depth regimes.

### Discordance between COI and nuclear loci

It is not surprising that mitochondrial COI exhibits stronger genetic divergence than the nuclear loci because its smaller effective population size should speed the effects of genetic drift and lineage sorting once gene flow is disrupted (e.g. [24,65,97]). Estimated mutation rates at our nuclear loci are on the same order of magnitude as that for COI, but these loci have not attained reciprocal monophyly. This pattern is expected to arise early in the process of speciation. Speciation, ongoing or recent, is often invoked in explaining discordance between nuclear and mitochondrial loci (e.g. [24,70,98-100]), and such evidence has been found recently in several marine taxa [52,54,58-60,101].

An alternative explanation for the stronger mitochondrial divergence is that either COI or another mitochondrial gene is under selection. Significantly negative neutrality indices (Tajima’s D and Fu’s Fs) were detected for some samples and can indicate purifying selection; however, these tests are highly sensitive to fluctuations in demographic parameters such as population size [102,103]. In particular, exponential population growth can cause negative neutrality indices, and indeed there is evidence for growth in *N* *. atacellana* , especially in the shallow population (Figure 5) where most of the significantly negative neutrality indices were detected. The McDonald-Kreitman test detected no selection on COI, but provides little insight into selection on other mitochondrial genes. Although we have no evidence of selection on COI, we cannot rule out the possibility that selection is operating on the mitochondrion and could account for the greater divergence at this locus (see [104,105]).

Significant fixed differences in COI are often ascribed to cryptic species, which are commonly revealed when morphologically identified species are analyzed genetically (e.g. [23,46,57,70,106,107]). In *N* *. atacellana* the four nuclear loci analyzed display high allelic diversity and some differentiation by depth; however, full sequences of the nuclear small ribosomal subunit (18S) and a 718 bp fragment of the large subunit (28S) were 100% identical among shallow and deep individuals (data not shown). Although not conclusive evidence, these results do suggest that populations have not been isolated long enough for divergence to accumulate in these more slowly evolving genes, indicating that populations of *N* *. atacellana* may be at a very early stage of species formation.

### What is disrupting gene flow across mid-rise depths?

The distance between the shallow and deep groups (100m depth, 40km distance) is almost certainly within the dispersal window of *N* *. atacellana* , which has demersal pelagic larvae that likely spend days to weeks dispersing [96,108]. The amphi-Atlantic distribution of *N* *. atacellana* [109] and the lack of genetic divergence across the North Atlantic [48] suggest dispersal distances are probably quite large, as has been found in other deep-sea taxa [110,111]. If connectivity between depth regimes is not limited by distance, then either hydrographic forces or selection (presumably at unsampled mitochondrial or nuclear loci) might be precluding gene flow.

The Deep Western Boundary Current (DWBC) flows south/southwestward in the immediate vicinity and depth of our sampled region, underneath and counter to the Gulf Stream [112,113], providing a possible isolating force to populations on either side. However, while the mean flow of the DWBC is southwest, highly complex small-scale variation is pervasive and probably more important for understanding actual particle trajectories and dispersal of largely passive invertebrate larvae. Drogues released at depth at three-month intervals over three years revealed significant submesoscale coherent vortexes (SCVs), long-lived eddies propagating from the DWBC and departing from its time-averaged southward trajectory [114]. The DWBC also interacts with the Gulf Stream, creating complex, variable, and non-isobathic water movements that could transport larvae from one side of the DWBC to the other [113-115]. Lagrangian simulations of particle releases in the DWBC show high potential for mixing and transport in the sampled region [115], making it unlikely that the DWBC is an effective barrier to gene flow. It is possible that larvae transported in SCVs from relatively cooler abyssal depths into warmer rise/slope depths or vice-versa face environmental challenges (see below) reducing or eliminating population connectivity through phenotype-environment mismatches (*sensu* [6,31]). It is also probable that the DWBC has waxed and waned through time in response to shifting climate [116,117] and was much stronger at times over the last 0.98 MY (i.e. through the glacial/interglacial cycles of the Pleistocene), possibly initiating the observed split in *N* *. atacellana* *.*

The lack of a clear isolating barrier and the extremely small scale over which divergence occurs suggest that selection might play an important role. A number of environmental gradients parallel changes in depth including temperature, oxygen, salinity, POC-flux, pressure, sediment characteristics, flow regimes, and topographic complexity as well as a suite of faunal characteristics such as the diversity, composition and trophic complexity of sediment communities [61-63]. Any of these gradients, singly or in combination, might lead to divergence, and they are frequently invoked as mediating adaptation (e.g. [118-120]), delimiting bathymetric distributions [62,121], or fostering population divergence and speciation [47,53,63,122,123]. Even weak environmental gradients can initiate divergence [38], and smoothly varying gradients can create sharply divergent taxa with deep phylogenetic splits [39]. In fact, the greater divergence at mitochondrial genes is exactly what we might expect if depth-related selection on mitochondria limited gene flow between depth regimes.

Identifying the precise environmental forces that shape bathymetric patterns of genetic variation will require considerably more research, but the greater divergence in COI compared to the nuclear loci is consistent with depth-related selection on mitochondrial variants. Metabolic processes might be especially sensitive to various depth-related environmental gradients (e.g. temperature, pressure, oxygen, etc.) leading to selection for different mitochondrial variants along the depth gradient. If this selection was strong enough to impede gene flow by selecting against migrants from contrasting depths (e.g. immigrant inviability [40]) it could account for the discordance between mitochondrial and nuclear loci as well as the greater divergence of COI.

A consensus is emerging for both shallow and deep organisms that strong differences among populations from different depths may be caused by environmental gradients that parallel depth (e.g. [37,47,48,54,59,101,124-130]). For example, depth related divergence between populations of the coral *Eunicea* *flexuosa* appears to be related to strong environmental selection against ecophenotypes from contrasting depths that reduces gene flow and may ultimately lead to speciation [37]. Similar inferences were made for another shallow-water Caribbean coral *Faviafragum* [124] and for several deep-water corals [101,125,129,131]. In the coral *Seriatopora* *hystrix* , reciprocal transplants of depth-segregated, genetically distinct ecotypes implicated post-settlement selection against migrants from parts of the reef formation at different depths [123,132]. Even pelagic species exhibit depth-related divergence that likely reflects environmental gradients that parallel depth [54]. A rapidly growing body of evidence suggests selection along environmental gradients can lead to speciation despite continued dispersal (reviewed in [6]).

Consistent with depth and its attendant environmental gradients playing an important role in diversification of deep-sea species, numerous studies have documented strong bathymetric divergence suggestive of cryptic species [55,57-60]. In addition, divergence is consistently much greater among populations separated vertically than those separated horizontally [45-51]. For example, genetic divergence in the amphipod *Eurythenesgryllus* was much greater across a 3.6 km depth gradient than across 4000km at the same depth [45] or even between the Atlantic and Pacific [46]. Finally, we often find closely related congeners separated bathymetrically (e.g. [52,54,109,130,131,133,134]), precisely the pattern that would emerge if species formation was frequently mediated by depth-related environmental gradients. Depth is the most frequently observed habitat difference between sibling species [23].

The environmental gradients imposed by increasing depth in the oceans make an intriguing parallel to altitudinal gradients in terrestrial systems, with greater depth analogous to greater altitude. Numerous studies have documented gradients of lower genetic diversity with increased altitude in plants (reviewed in [135], also [136-138]) and animals (reviewed in [139], also [140-142]). While correlations in terrestrial systems are not always negative or linear, they are frequently accompanied by significant differentiation of highland and lowland clades [143-147], and often implicate the greater importance of vertical vs. horizontal distance. This was exactly the pattern documented in a widespread passerine bird in the Peruvian Andes, which was attributed to altitudinal shifts in selection on mitochondrial variants [148]. There is some evidence that, at least for animals, increased hypoxia at high altitude drives genetic differentiation and isolation-by-altitude [147-149], although adaptive changes in reproductive characteristics have also been found [150].

Another possible explanation for the depth divergence is that it formed in allopatry and the shallow and deep groups experienced secondary contact in the western North Atlantic, resulting in differential introgression of mitochondrial and nuclear genes. However, *N* *. atacellana* is widely distributed in the Atlantic, with virtually no divergence between the eastern and western North Atlantic and only modest divergence between the North and South Atlantic [48]. In addition, a similar depth divergence occurs within the Argentine basin but involves different haplotypes. Unfortunately, only formalin-fixed samples are available for the South Atlantic, restricting genetic analyses to mitochondrial loci. We cannot exclude the possibility that divergence was allopatric, but pan-Atlantic phylogeographic analyses indicate the greatest divergence is between shallow and deep groups in the western North Atlantic.

The deep ocean is a vast semi-continuous ecosystem that supports a highly diverse and largely endemic fauna. The evolutionary processes that gave rise to this distinctive fauna, the spatial and temporal scales over which they operate, and the geography and bathymetry of divergence are poorly understood. Given the limited ecological opportunity and the lack of obvious mechanisms that would allow population differentiation and speciation, it is unclear how new species form, especially at a rate sufficient to explain the high levels of diversity. Unraveling how and where evolution unfolds is critical for explaining biogeographic patterns of diversity [63], predicting how deep-sea ecosystems might respond to climate change [151-153], developing conservation and management strategies to mitigate the intense exploitation of deep-sea resources [64,154] and identifying appropriate locations and scales for MPAs [155,156]. Widespread and consistent divergence across depth gradients suggest depth and its concomitant environmental gradients may provide one of the primary mechanisms mediating population differentiation and speciation, especially below the continental shelves.

## Supporting Information

Figure S1 **Haplotype network for COI.** Circle size indicates number of individuals possessing that haplotype. Small circles represent unsampled haplotypes required to connect the network. Squares indicate the most likely ancestral haplotype. Haplotypes are shaded shallow and deep as in Figure 1.
(TIF)

Figure S2 **Haplotype network for CAL.** Haplotype shape, size, and coloring are as in Figure S1.
(TIF)

Figure S3 **Haplotype network for MAC.** Haplotype shape, size, and coloring are as in Figure S1.
(TIF)

Figure S4 **Haplotype network for DAC3.** Haplotype shape, size, and coloring are as in Figure S1.
(TIF)

Figure S5 **Haplotype network for DAC6.** Haplotype shape, size, and coloring are as in Figure S1.
(TIF)

Figure S6 **Population demographic history and migration estimates from IM for nuclear loci.** The gray box indicates the estimated effective population size (Ne) of the ancestral population. Estimated splitting time is indicated by the horizontal line. Descendant shallow and deep populations are represented above the line by polygons whose starting width is the estimated Ne just after the split and whose upper width is the estimated contemporary Ne. Curved dotted arrows represent estimated migration rates per generation, forward in time from source to destination. Coloring of shallow and deep is as in Figure 1.
(TIF)

Table S1 **PCR reaction mixtures and thermocycler conditions for amplified loci.** PCRs were performed in 50µL reactions consisting of 1X GoTaq Flexi buffer with loading dye (Promega, Madison, WI), 2.5mM MgCl_2_, 2pmol dNTPs, 1.2pmol of each primer, 2µL genomic DNA, and 1 U of Taq polymerase (Promega). Conditions specific to each locus are given below; all protocols had an initial denaturation of 94°C for 3 min., 35 cycles of (denaturation at 94°C for 30 sec., annealing at the indicated temperature for 45 sec., extension at 72°C for 1 min), final extension at 72°C for 3 min., and a final hold at 4°C.
(DOC)
