# Supplementary figures and images for: Population Differentiation and Species Formation in the Deep Sea: The Potential Role of Environmental Gradients and Depth
Source: PLoS One. 2013 Oct 1;8(10):e77594. doi: 10.1371/journal.pone.0077594 (PMC3788136; doi:10.1371/journal.pone.0077594)

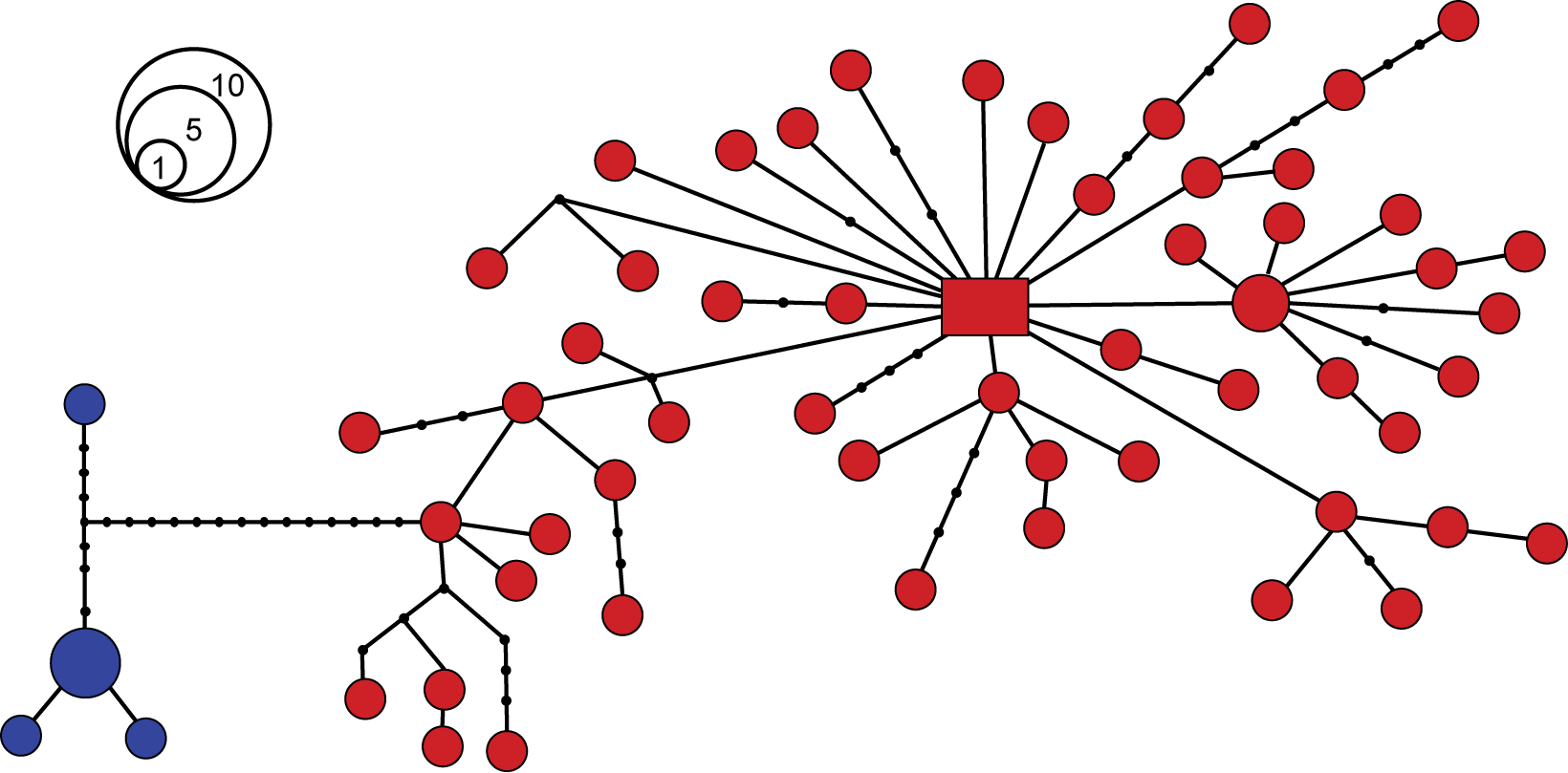

Supplement: Figure S1 — Haplotype network for COI. Circle size indicates number of individuals possessing that haplotype. Small circles represent unsampled haplotypes required to connect the network. Squares indicate the most likely ancestral haplotype. Haplotypes are shaded shallow and deep as in Figure 1. (TIF) [file pone.0077594.s001.tif]

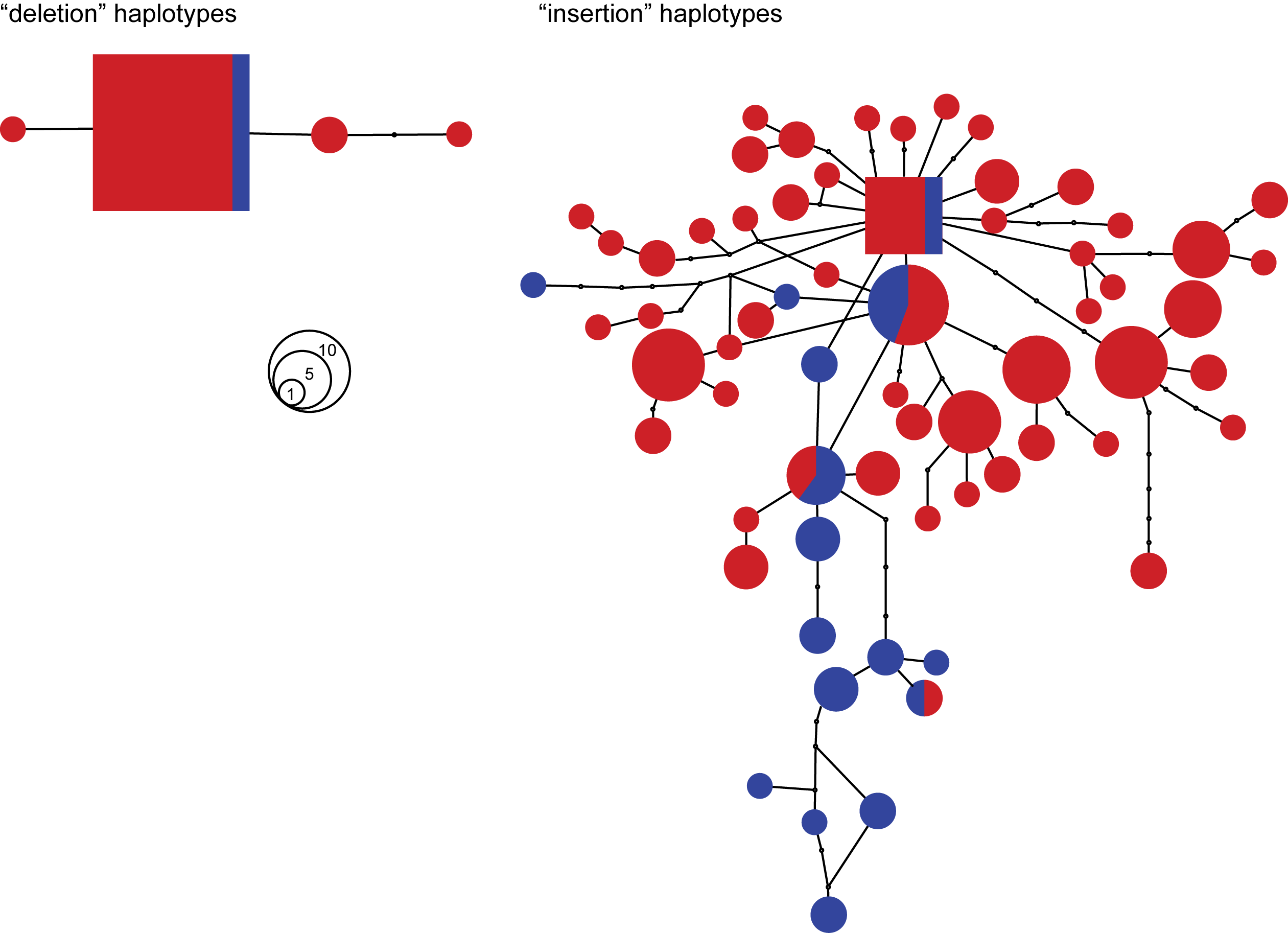

Supplement: Figure S2 — Haplotype network for CAL. Haplotype shape, size, and coloring are as in Figure S1. (TIF) [file pone.0077594.s002.tif]

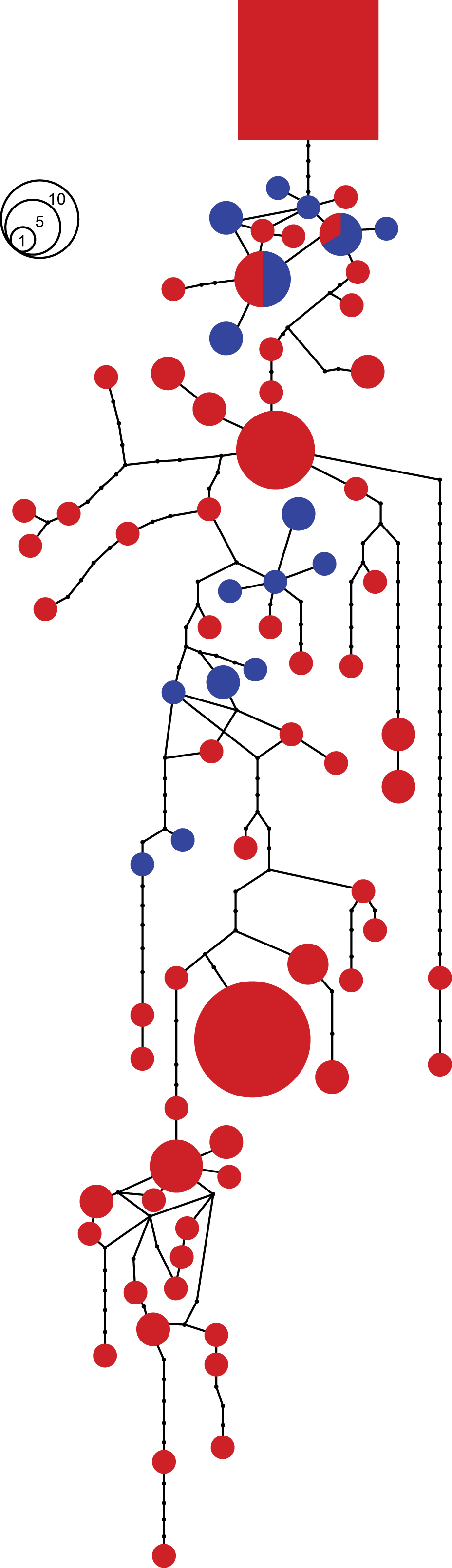

Supplement: Figure S3 — Haplotype network for MAC. Haplotype shape, size, and coloring are as in Figure S1. (TIF) [file pone.0077594.s003.tif]

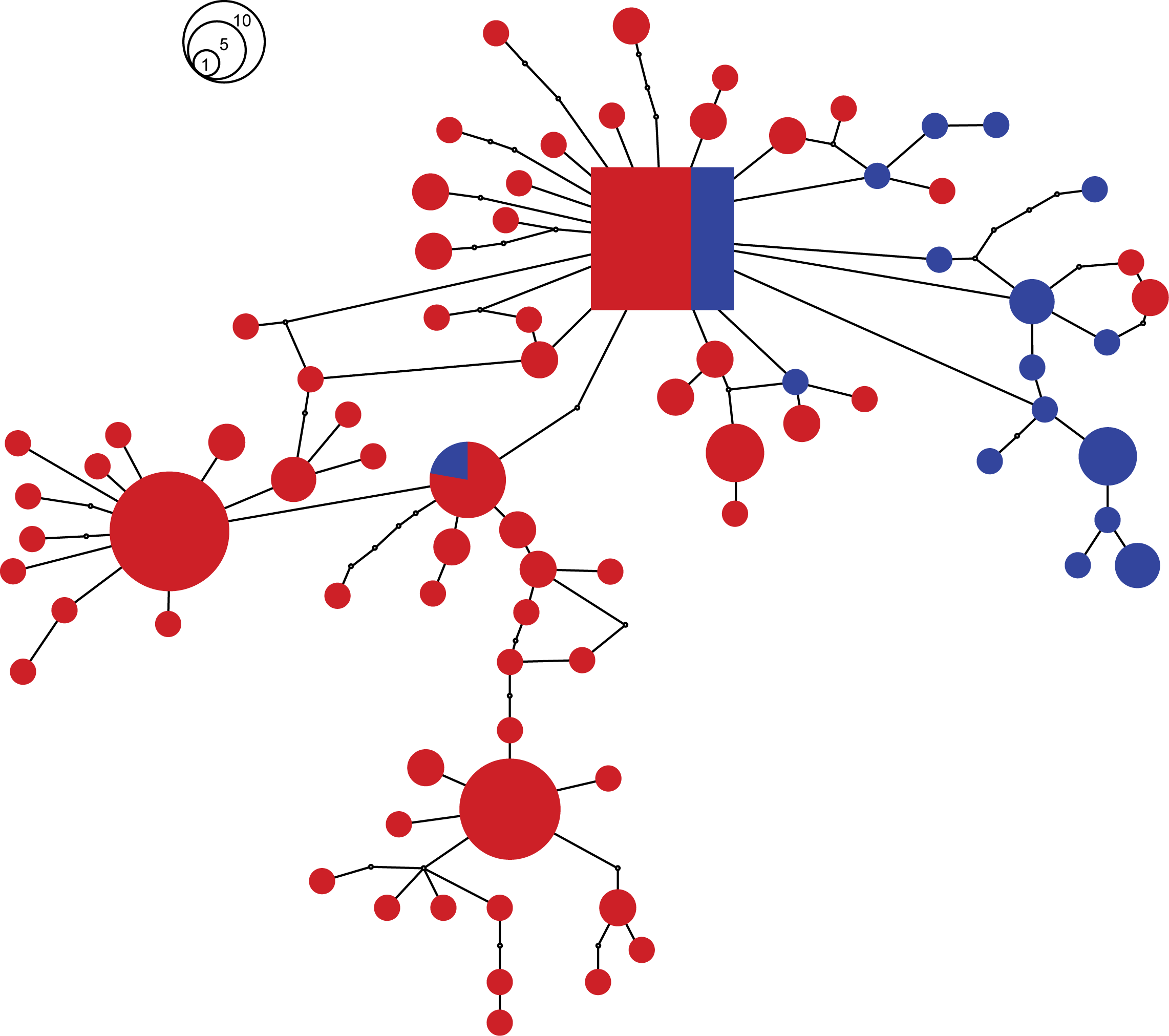

Supplement: Figure S4 — Haplotype network for DAC3. Haplotype shape, size, and coloring are as in Figure S1. (TIF) [file pone.0077594.s004.tif]

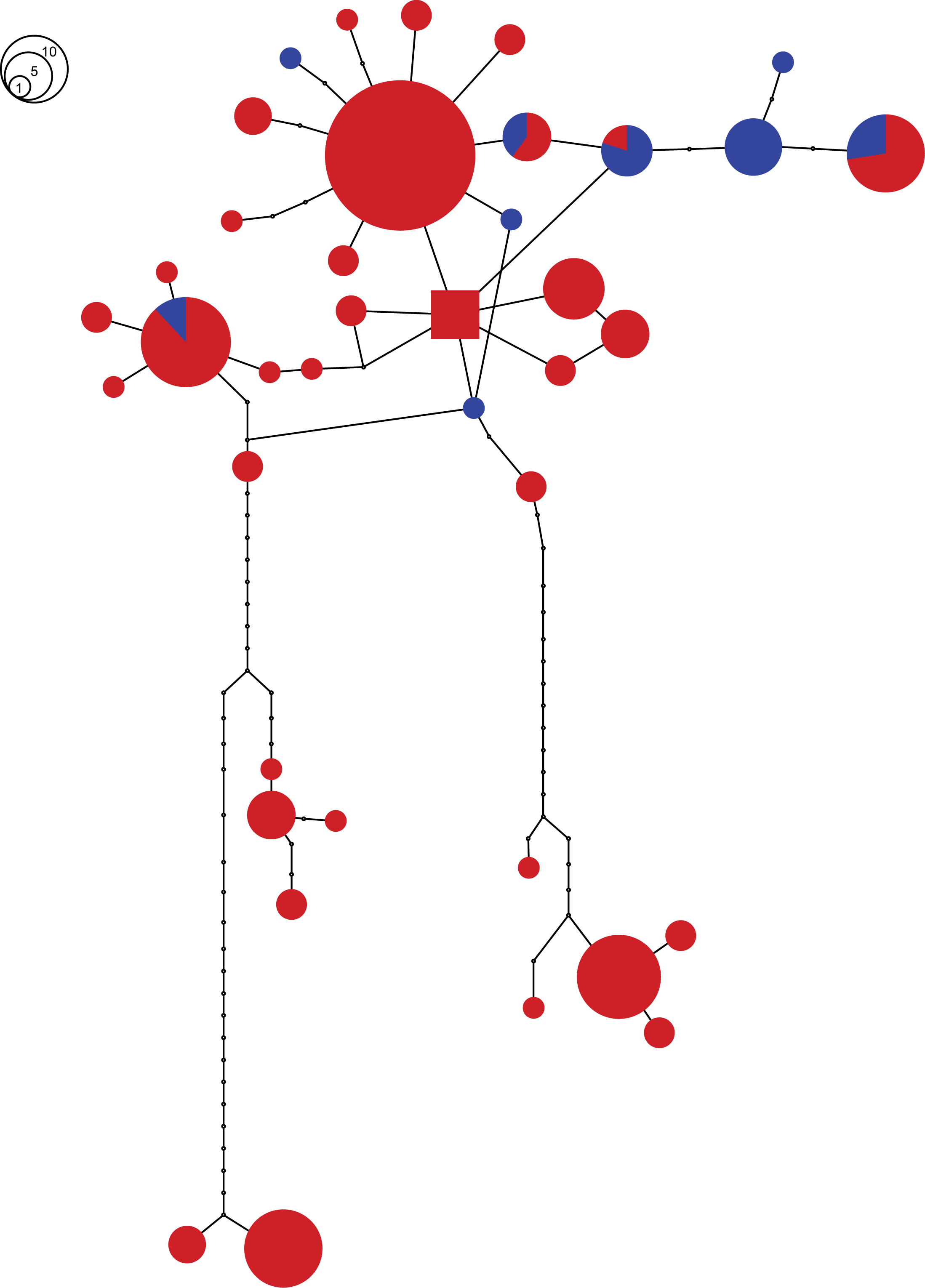

Supplement: Figure S5 — Haplotype network for DAC6. Haplotype shape, size, and coloring are as in Figure S1. (TIF) [file pone.0077594.s005.tif]

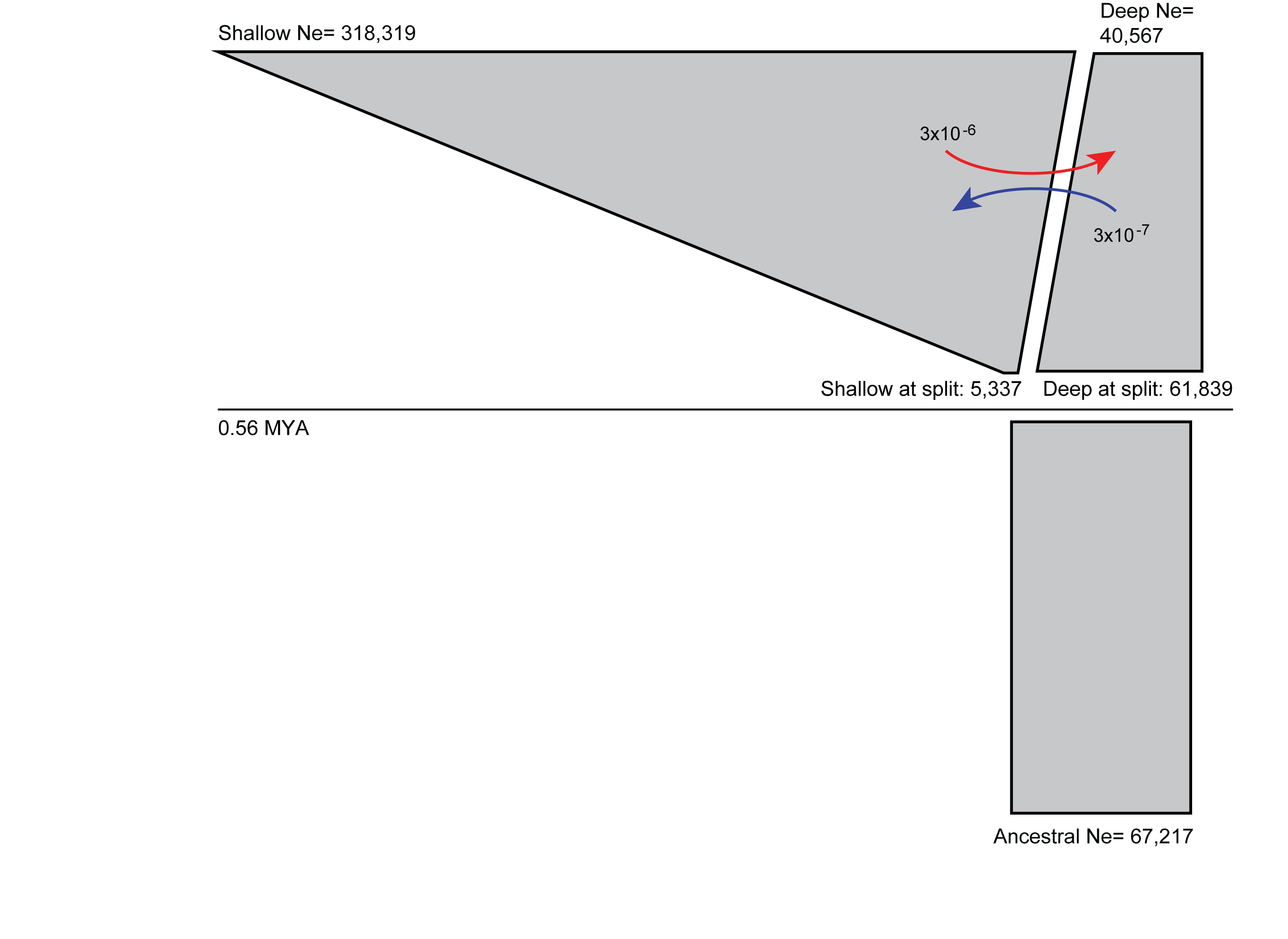

Supplement: Figure S6 — Population demographic history and migration estimates from IM for nuclear loci. The gray box indicates the estimated effective population size (Ne) of the ancestral population. Estimated splitting time is indicated by the horizontal line. Descendant shallow and deep populations are represented above the line by polygons whose starting width is the estimated Ne just after the split and whose upper width is the estimated contemporary Ne. Curved dotted arrows represent estimated migration rates per generation, forward in time from source to destination. Coloring of shallow and deep is as in Figure 1. (TIF) [file pone.0077594.s006.tif]
