# Supplementary material for: Population Differentiation and Species Formation in the Deep Sea: The Potential Role of Environmental Gradients and Depth
Source: PLoS One. 2013 Oct 1;8(10):e77594. doi: 10.1371/journal.pone.0077594 (PMC3788136; doi:10.1371/journal.pone.0077594)
Supplement: Table S1 — PCR reaction mixtures and thermocycler conditions for amplified loci. PCRs were performed in 50µL reactions consisting of 1X GoTaq Flexi buffer with loading dye (Promega, Madison, WI), 2.5mM MgCl2, 2pmol dNTPs, 1.2pmol of each primer, 2µL genomic DNA, and 1 U of Taq polymerase (Promega). Conditions specific to each locus are given below; all protocols had an initial denaturation of 94°C for 3 min., 35 cycles of (denaturation at 94°C for 30 sec., annealing at the indicated temperature for 45 sec., extension at 72°C for 1 min), final extension at 72°C for 3 min., and a final hold at 4°C. (DOC) [file pone.0077594.s007.doc]

| Locus | Nesting round | Primers | Sequence (5'–3') | Annealing (°C) |
| --- | --- | --- | --- | --- |
| COI | 1 | LCO1490 | GGTCAACAAATCATAAAGATATTGG | 45 |
|  |  | HCOoutout | GTAAATATATGRTGDGCTC |  |
|  | 2 | LCO1490 | GGTCAACAAATCATAAAGATATTGG | 45 |
|  |  | HCO2198 | TAAACTTCAGGGTGACCAAAAAATCA |  |
| CAL | – | DaCALifF2b | ATCATCCACTCTTCRTGAGAG | 50 |
|  |  | DaCALifR2b | TGAAAAAATACTGATTCAGATAGG |  |
| MAC | – | DaMACifF1 | ATAGTAAAGACGTTGTGCTTACG | 50 |
|  |  | DaMACifR2 | CATAYTTGTATAACACTTTAACG |  |
| DAC3 | – | DAC3f | TTCACGCGATTCACAAACTAA | 54 |
|  |  | DAC3r | GAACGTCTGGGGGTTGACTA |  |
| DAC6 | 1 | DAC6f | TGATGGGAAACCAACTACCC | 54 |
|  |  | DAC6r | GGCCTGTCTGGCCTGCTA |  |
|  | 2 | DAC6f2 | TCGTTATTCGAAATGGCTGA | 49 |
|  |  | DAC6r2 | CCGACACTGAGGTACCGATT |  |
